# Genomic Signatures of Adaptive Evolution in *Taenioides* sp. During Northward Invasion

**DOI:** 10.3390/ijms26199613

**Published:** 2025-10-01

**Authors:** Kun Huang, Tianwei Liu, An Xu, Jing Yu, Yijing Yang, Jing Liu, Fenghui Li, Denghui Zhu, Li Gong, Liqin Liu, Zhenming Lü

**Affiliations:** National Engineering Laboratory of Marine Germplasm Resources Exploration and Utilization, College of Marine Sciences and Technology, Zhejiang Ocean University, Zhoushan 316022, China

**Keywords:** genomic resequencing, adaptive evolution, northward invasion, *Taenioides* sp.

## Abstract

The success and impact of biological invasions depend on adaptations to novel abiotic and biotic selective pressures. However, the genetic mechanisms underlying adaptations in invasive species are inadequately understood. *Taenioides* sp. is an invasive worm goby, originally endemic to brackish waters in the estuaries of Southeastern China, and now colonizes multiple inland freshwaters of North China within decades as a byproduct of the East Route of South-to-North Water Transfer (ESNT) project. However, the molecular mechanisms underlying their adaptations to the climate of North China, especially the temperature regime, are unknown. Here, we performed genomic resequencing analysis to assess genetic diversity and population genetic structure, and further investigated the genomic signatures of local adaptation in the invasive population of *Taenioides* sp. during their northward invasion. We revealed that all invasive populations exhibited no genetic differentiation but low gene flow and an obvious signal of population bottleneck. Yangtze River estuary may serve as the source population, while Gaoyou Lake serves as a potential bridgehead of the invasion. Selective sweep analyses revealed 117 genomic regions, containing 673 candidate genes, under positive selection in populations at the invasive front. Redundancy analysis suggested that local temperature variables, particularly the monthly minimum temperature, represent critical evolutionary forces in driving adaptive divergence. Functional enrichment analyses revealed that multiple biological processes, including metabolism and energy production, substance transmembrane transport, and neural development and synaptic transmission, may play important roles in adaptation to regional temperature conditions. Our findings revealed a scenario of adaptive evolution in teleost species that underpins their regional climate adaptation and successful establishment of invasive populations in a human-facilitated invasion context. Proper management strategies should be established to manage *Taenioides sp* invasion as soon as possible.

## 1. Introduction

Invasive species are a threat to native ecosystems, and can exert a rapid and severe impact on global biosafety, food security, and human health [1]. Understanding the causes of biological invasions may serve as a prerequisite to mitigate the impact of invasive species [2], but it is usually complex and requires detailed parallel field studies conducted in both native and introduced ranges to investigate how biogeographical shifts alter individual performance, population success, and the community-level impacts of the invading species [3]. An alternative step may lie in comprehending the demographic or evolutionary determinants underlying the successful establishment of invasive species, which would enable our improved predictability of their invasive potential and establish enhanced management strategies for their effective control [4]. Although several ecological or demographic determinants are known as important factors underlying the successful colonization and establishment of populations, including propagule pressure or plasticity in life history (e.g., faster growth, or younger age at first maturity) [5,6], other evolutionary factors, such as drift, selection, or adaptive phenotypic plasticity in this process, are still limitedly known [4]. However, well-documented cases have been recorded, including that of the invasive marine vertebrates cornetfish (*Fistularia commersonii*) [2] and invertebrate ascidian (*Molgula manhattensis*) [7], where the rapid evolution of adaptive loci or genomic regions facilitated rapid range expansion and invasion success. Fortunately, with the rapid progress of next-generation sequencing (NGS) technology, the use of genomic tools in invasion contexts has gained increasing recognition, and the provision of reference genomes of more and more non-model species is allowing for the accurate estimation of the role of pre- and post-introduction genetic variation, demographic history, and adaptive potential in invasion success from a genomics perspectives [3]. However, to date, the use of genomics in the context of biological invasion is still in its infancy, thus leaving many unanswered questions about the adaptive processes involved in successful invasions and associated ecological and evolutionary consequences.

*Taenioides* sp. is an invasive worm goby originally known to inhabit muddy bottoms of brackish waters in coastal areas of southeast China [8,9]. The estuary areas around Yangtze River and Lüsi fishing ground almost form the northernmost limits of its natural distribution, according to the historical records [10,11]. However, it has begun to invade northward recently, and successfully colonized multiple inland freshwater lakes in North China and caused severe damage to aquatic ecosystems [8,9]. The northward freshwater invasion of *Taenioides* sp. was hypothesized to be facilitated by the East Route of the South-to- North Water Transfer (ESNT) project, which pumps water from the lower reaches near the Yangtze River estuary and diverts it northward to resolve the water shortage problem in north China persisting since the 1960s [8,9]. The diverted water flow through several large lakes of North China, including Gaoyou Lake (GYL), Hongze Lake (HZL), Luoma Lake (LML), Nansi Lake (NSL), and finally reaching Dongping Lake (DPL) in Shandong province [8], makes these lakes the most affected areas of the invasion, except for Dongping Lake, where no colonization of *Taenioides* sp. populations has been recorded to date [8]. The establishment of invasive *Taenioides* sp. populations in these lakes occurred at an extremely rapid rate: it was first recorded in Gaoyou and Hongze lakes in the 1980s [8,10,11], and increased abundance and northward dispersal into Luoma Lake in 2005, and invaded further northward into NSL, and was first reported in the lower stream of NSL, Weishan Lake (WSL) in 2011, and gradually observed to colonize the upper stream of NSL, Zhaoyang Lake (ZYL) in 2014 [8]. As a result, within around 60 years since the 1960s, the distribution of *Taenioides* sp. in its invasive range has spanned the tropic and subtropic areas of Southeast China to the temperate areas of Shandong Province in North China. Such a northward invasion scenario of *Taenioides* sp. has offered a unique opportunity to investigate how the adaptive process has contributed to the successful colonization, regional expansion, and hence the invasion success of an invasive species. However, recent molecular studies on *Taenioides* sp. populations have mainly focused on characterizing the invasive origin and dispersal routes facilitated by the ESNT project [8,9]. All these attempts have primarily utilized only a few molecular markers, including mitochondrial [8] and microsatellite DNA [9]. To date, no adaptive process, including selection and adaptive phenotypic plasticity, has been characterized in invasive populations of *Taenioides* sp., aiming to reveal the molecular mechanism underlying their invasion success from genetic signatures, especially from a genomic point of view.

In the present study, based on our previously assembled genome of *Taenioides* sp., we performed whole-genomic resequencing on specimens collected from six invasive and native populations around the affected areas of ESNT projects. We also added two additional native populations around these areas as representatives of the potential invasive sources of the invasion. By performing the population genomics analysis of *Taenioides* sp. from its native and invasive regions, our study aimed to (1) systematically investigate the invasion process and hypothetical dispersal in the invasive range, which provides an evolutionary framework for better understanding the genetic adaptation of *Taenioides* sp. during their northward invasion; and (2) identify candidate loci under selection to determine their role in the northward invasion success of *Taenioides* sp. Our results could provide insights into how adaptive processes facilitate the successful establishment and range expansion of invasive species and thus, provide important information for establishing proper management strategies for their effective control.

## 2. Results

### 2.1. SNP and Genetic Diversity

**Figure 1:**
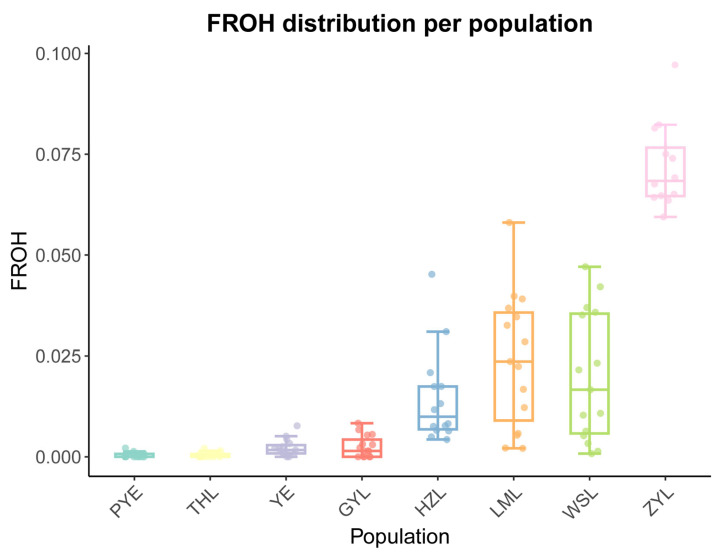
Genomic inbreeding coefficients inferred from the proportion of the genome within ROH (F_ROH_). The y-axis represents the F_ROH_ value for each population. ZYL, WSL, LML, HZL, GYL, YE, THL, and PYE in the x-axis represent samples collected from Zhaoyang Lake, Weishan Lake, Luoma Lake, Hongze Lake, Gaoyou Lake, Yangzhi River Estuary, Thaihu Lake, and Pingyang Aojiang Estuary, respectively. The minima, maxima, center, and the upper and lower bounds of the box represent the maximum, minimum, median value, and upper and lower quartiles, respectively.

A total of 116 fish were used, and their genomes were resequenced in the study. On average, 1.50 × 10^8^ clean reads were yielded for each specimen and then mapped to the assembled reference genome of *Taenioides* sp. The mean coverage of each site was calculated, and revealed a mean depth of 29.84× (23.07~68.34×) in the mapped regions of all the samples. After a strict filtration and quality control process, a total of 6,365,664 high-quality SNP sites were generated across all samples and thereby used for the downstream analyses. Genome-wide estimates of diversity varied substantially between the native and invasive populations. In general, native populations showed much higher diversity in all statistical parameters, such as percentage of polymorphic loci (%), heterozygosity observed (Ho), heterozygosity expected (He), and nucleotide diversity (Pi), than invasive populations (Table 1), consistent with the “source population richness” phenomenon usually observed in the invasion context [4,12]. More specifically, the PYE population generally harbored the highest diversity in all parameters examined among the three native populations, while the YE population displayed the lowest. The ZYL population showed the lowest diversity in all parameters estimated among the five invasive populations, and was even the lowest across the regions, while GYL exhibited the highest diversity among the five invasive populations (Table 1). Accordingly, the genomic inbreeding coefficient (F_ROH_) is generally higher in invasive populations than their native counterparts, with the ZYL population displaying the highest F_ROH_ value and the PYE population showing the lowest among all the studied populations (Figure 1). These results were, again, in line with the fact that the ZYL population represents the northernmost front of the invasion and likely experiences the greatest bottleneck events during the invasion.

### 2.2. Population Genetic Structure

**Figure 2:**
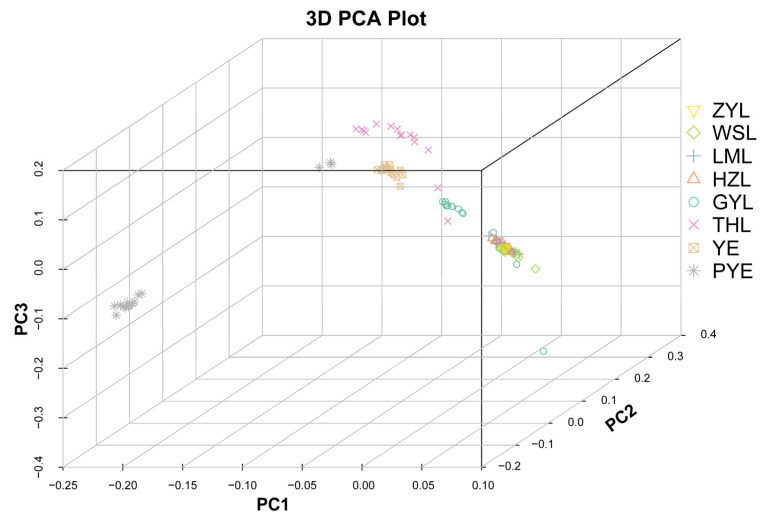
Principal component analysis (PCA) showing genetic distance among samples of *Taenioides* sp. Sampling sites are colored according to the population to which they belong. Individuals are labeled according to the location of sampling as coded in Table 1 (ZYL, WSL, LML, HZL, GYL, YE, THL, and PYE represent samples collected from Zhaoyang Lake, Weishan Lake, Luoma Lake, Hongze Lake, Gaoyou Lake, Yangtze River Estuary, Thaihu Lake, and Pingyang Aojiang Estuary, respectively).

**Figure 3:**
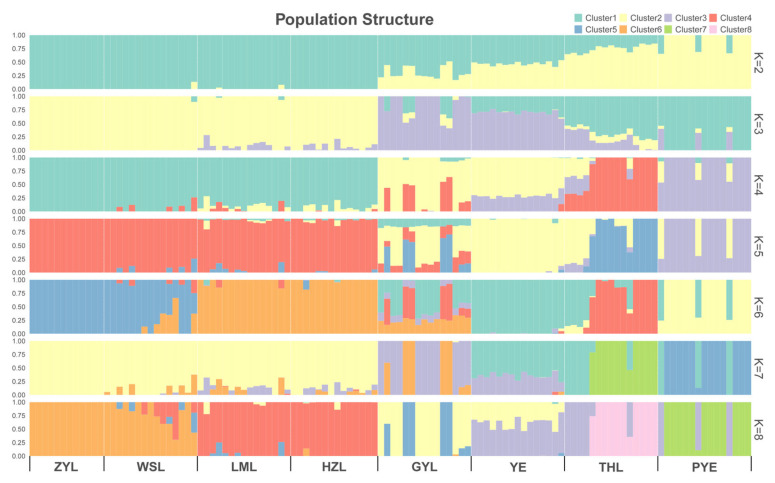
Genetic structure of *Taenioides* sp. populations within their native and invasive ranges inferred using ADMIXTURE. Each bar represents an individual, with different colors corresponding to one of the K ancestry clusters and length proportional to the assignment to that particular cluster. Individuals are grouped according to the location of sampling (ZYL, WSL, LML, HZL, GYL, YE, THL, and PYE represent samples collected from Zhaoyang Lake, Weishan Lake, Luoma Lake, Hongze Lake, Gaoyou Lake, Yangzhi River Estuary, Thaihu Lake, and Pingyang Aojiang Estuary, respectively).

**Figure 4:**
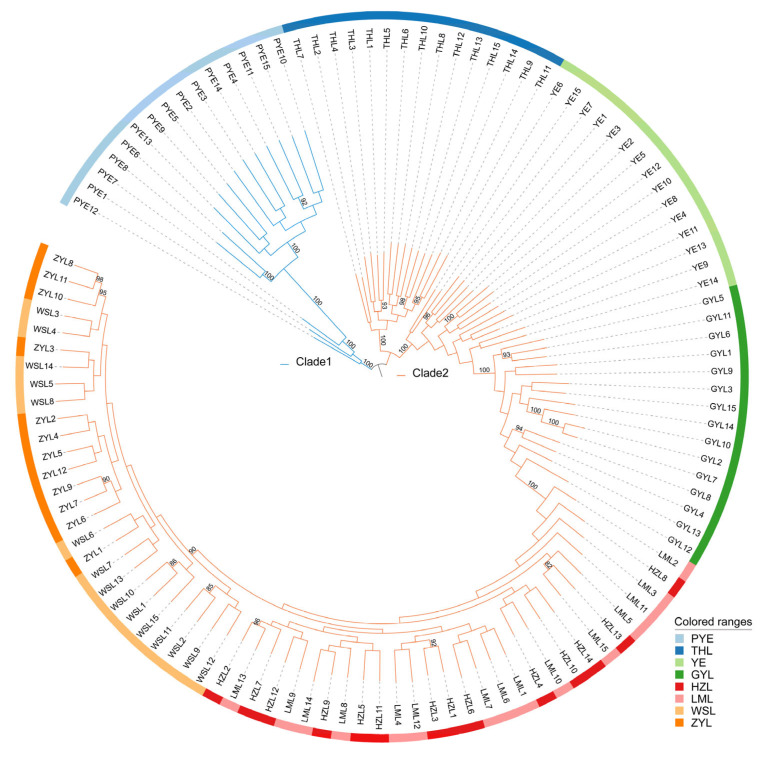
Maximum likelihood (ML) trees were constructed from the allele-shared matrix of SNPs among *Taenioides* sp. populations. Sampling sites are colored according to the population to which they belong. Individuals are labeled according to the location of sampling as coded in Table 1 (ZYL, WSL, LML, HZL, GYL, YE, THL, and PYE represent samples collected from Zhaoyang Lake, Weishan Lake, Luoma Lake, Hongze Lake, Gaoyou Lake, Yangzhi River Estuary, Thaihu Lake, and Pingyang Aojiang Estuary, respectively).

**Figure 5:**
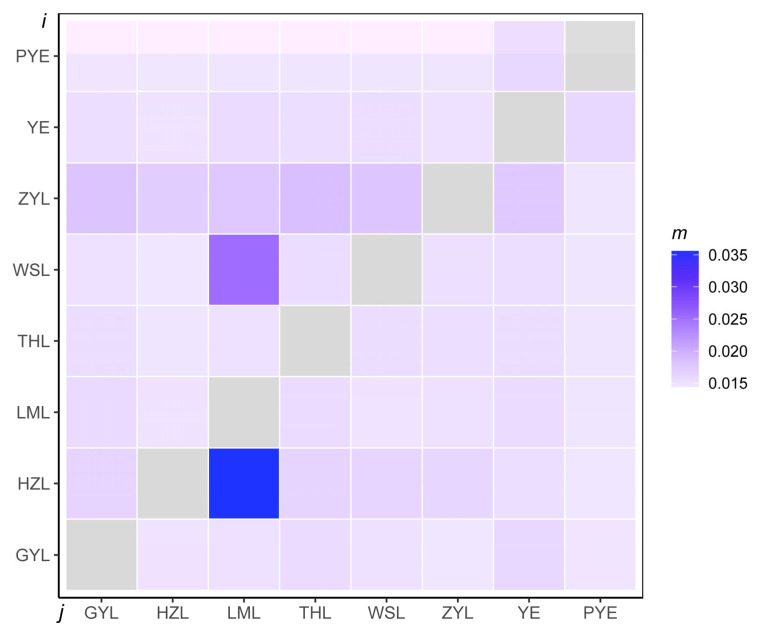
Recent gene flow among eight sampling localities for *Taenioides* sp. based on genomic resequencing. The gene flow direction was from population j–i, as indicated in x- and y-axes. Dark and light colors indicate a relatively high and low level of gene flow, respectively. Recent gene flow was represented by the migration rate (m) calculated from BayesAss. ZYL, WSL, LML, HZL, GYL, YE, THL, and PYE represent samples collected from Zhaoyang Lake, Weishan Lake, Luoma Lake, Hongze Lake, Gaoyou Lake, Yangzhi River Estuary, Thaihu Lake, and Pingyang Aojiang Estuary, respectively.

Estimates of divergence among the 8 populations showed a genome-wide Fst of 0.021–0.129 across all autosomal SNPs (Table 2), suggesting low-to-moderate differentiation among the populations. Such differentiation among the populations resulted in a weak but significant population genetic structure, according to our downstream population structure analyses. Our principal component analyses (PCA) divided all genotyped samples into two specific clades, with the first two components explaining 9.96% and 5.74% of total genetic variance, respectively, according to a Tracy–Widom test (Figure 2). The first clade mainly comprised individuals from PYE populations, and the second clade primarily comprised individuals from the remaining seven populations. Such population substructure was also confirmed by the results of our admixture analyses, which also subdivided all genotyped samples into two specific clades when the number of clusters (K) was 2, i.e., optimal (Figure 3; Supplemental Figure S1). Among them, one clade was from all individuals of the PYE population, while the other clade was from the remaining seven populations. Further population substructuring was observed when K = 3, in which individuals of THL, YE, and GYL were subdivided from Clade 2 and formed a separate clade, indicating their genetic affinity between these native and invasive populations. When K = 4, the THL population was further divided from YE and GYL populations, and formed a specific clade. The YE population could be only divided from the GYL population when K = 5, also indicating their close relationship in genetic composition (Figure 3). When K = 6, individuals of HZL and LML were further subdivided from WSL and ZYL populations, and again formed a specific separate clade. A maximum likelihood (ML) tree further lent support for these differentiation patterns, with PYE (Clade 1) and the remaining 7 populations (Clade 2) reflecting the grouping of populations with high bootstrap support (100%) (Figure 4). Clade 2 included most of the (7/8) populations analyzed in this study, in which the five invasive populations of GYL, HZL, LML, WSL, and ZYL were more closely related to the native population of YE, other than THL, with YE forming the base group of the tree (Figure 4). These results indicated that YE may serve as the invasion source of the alien *Taenioides* sp. populations, which has also been implicated in previous studies [8,9]. However, as far as the five invasive populations were concerned, individuals of GYL more genetically resembled those of the source population YE than any other population, while individuals of HZL and LML most likely came from the GYL population, and individuals of WSL and ZYL formed the most specialized group, which likely came from the HZL and LML populations, as inferred from the ML tree (Figure 4). Such substructuring of the invasive populations accorded well with what should be expected from an invasion process through the stepping-stone dispersal model [8], possibly reflecting their historical invasion dynamic and model of gene flow among invasive populations. However, such substructuring of the invasive populations may predict limited gene flow among populations. To test our inference, an AYESASS analysis was further employed to estimate the contemporary gene flow among the native and invasive populations, and the results indicated that except for a relatively higher rate of gene flow being detected among four invasive populations (ZYL, WSL, LML, and HZ), a general low level of gene flow was found among all the native and invasive populations (Figure 5) studied.

### 2.3. Candidate Loci Under Selection

**Figure 6:**
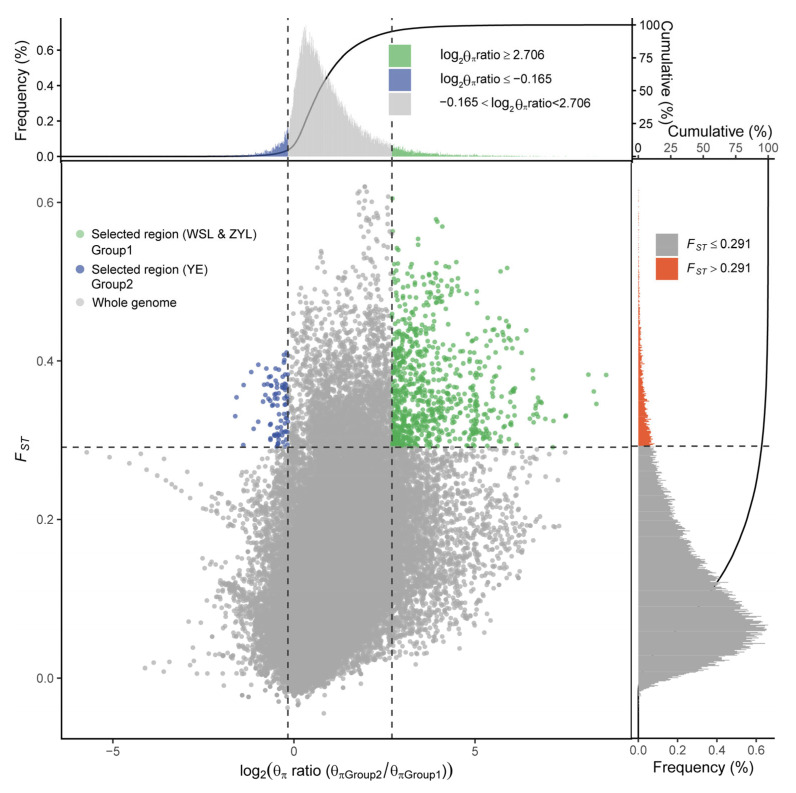
Distribution of log2 (Pi) ratios and Fst values, which were calculated in 100 kb windows sliding in 10 kb steps. Data points located to the left and right of the vertical dashed lines (corresponding to the 5% left and right tails of the empirical log2 (Pi) ratio distribution), and above the horizontal dashed line (the 5% right tail of the empirical FST distribution) were identified as selected regions for populations at the invasive front (green points).

**Figure 7:**
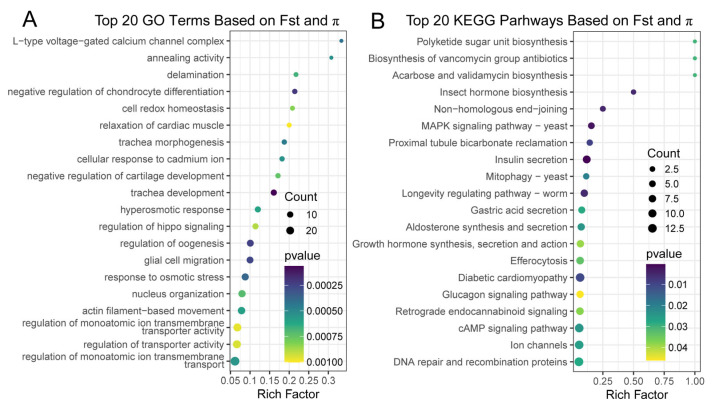
Go (**A**) and KEGG (**B**) enrichment results of candidate genes under selection identified in populations at the invasive front by selective sweeps. Only top 20 GO terms and KEGG pathways enriched from the candidate genes under selection are shown. The rich factor represents the ratio of differentially expressed genes to the total number of genes in each pathway. The color of the dots indicates the significance level (*p*-value) of enrichment, and the size of the dots represents the number of genes enriched in each pathway.

To identify the possible adaptive selection they may experience during their northward invasion, as expected from the heterogeneous population structure and the latitude gradients they span during the northward invasion, we first estimated the strength of positive selection or the rate of adaptive substitution (α) in *Taenioides* sp. populations using the standard McDonald–Kreitman test. The results revealed a comparatively low rate of adaptive substitution in invasive populations compared to their native counterparts (except for THL) (Table 3), possibly attributed to their potentially smaller population size (Ne). When the selection strength was compared within the invasive populations, the α value generally increased with latitude with the northernmost invasive populations of ZYL, SL, and LML possessing a higher rate of adaptive substitution despite their genetic bottleneck effect, indicating the possible adaptive selection they may experience during their northward invasion. To better capture these signatures of selection in invasive populations during their northward invasion, we selected ZYL and WSL at the invasive front as representatives of invasive populations, and YE as the source population to perform selective sweep analyses by using both metrics of Fst and pairwise nucleotide diversity (Pi). Our results identified 117 genomic regions displaying signatures of selection, containing 673 candidate genes under positive selection in WSL and ZYL populations (Figure 6; Supplemental Table S2). Functional enrichment (GO) analysis indicated that the top positively selected genes were significantly enriched in multiple biological processes, such as “regulation of hippo signaling”, “cell redox homeostasis”, “response to osmotic stress”, “regulation of oogenesis”, and “actin filament-based movement” (Figure 7A; Supplemental Table S3). KEGG enrichment analysis indicated that signal pathways such as “Insulin secretion (ko04911)”, “Glucagon signaling pathway (ko04922)”, “Diabetic cardiomyopathy (ko05415)”, “Polyketide sugar unit biosynthesis (ko00523)”, “Ion channels (ko04040)”, “Aldosterone synthesis and secretion (ko04925)”, “Proximal tubule bicarbonate reclamation (ko04964)”, “DNA repair and recombination proteins (ko03400)”, “Retrograde endocannabinoid signaling (ko04723)”, and “Growth hormone synthesis, secretion and action (ko04935)” were significantly enriched (*p* < 0.05) (Figure 7B; Supplemental Table S4), which suggested the crucial role of nutrient metabolism and energy production, osmotic regulation, DNA repairs, and hormone production and secretion in regional environmental adaptation during their northward invasion of *Taenioides* sp.

### 2.4. Gene–Environment Associations

To understand the possible role of regional temperature regime in shaping genetic differentiation and adaptive evolution among *Taenioides* sp. populations, we further performed an environmental association analysis based on RDA analyses. According to environmental correlation analyses (R^2^ > 0.7), only two water temperature variables, thetao_ltmin and thetao_mean, were preserved from the initial 6 parameters for subsequent analysis (Supplemental Figure S2). Our results indicated that the RDA was globally significant (*p* < 0.001) and explained about 3.32% of the total variation (adjusted R^2^ = 0.033). Among the two RDA axes analyzed, only RDA1 axes were significant (*p* =  0.001), and both of the temperature variables retained in RDA were significant (*p* =  0.001) as explanatory variables (Supplemental Figure S2). Considering the significantly constrained canonical axes, 11,487 unique candidate loci were finally identified, and the majority of the detected SNPs (6820; 59.37%) were strongly correlated with thetao_ltmin. The remaining candidate loci (4667; 40.63%) were associated with thetao_mean. Thetao_ltmin explained the largest proportion of RDA detections, suggesting that the minimum temperature range explained more allelic variation than any other temperature variable, and may likely play important roles in driving the genomic landscape evolution and hence regional climate adaptation of invasive *Taenioides* sp. populations.

### 2.5. Functional Annotation and Enrichment

Combining both data matrixes of RDA and selective sweep analyses, 1705 candidate SNPs were identified to be positively selected in overlap, corresponding to 51 candidate genes under positive selection (Supplemental Table S5). These positively selected genes were primarily associated with multiple biological processes, such as metabolism and energy production (*G6PD*, *SLC25A14*, *CEL*, *PGS1*), substance transmembrane transport (*COG5*, *GOLT1B*), cytoskeletal structure and organization (*EFEMP1*, *EML6*, *TPRN*), and neural development and synaptic transmission (*SCN8A*, *CACNA2D1*, *CACNA1D*, *KCNQ2*, *SEMA3D*), implying their critical roles in adaptation to regional temperature conditions, and hence the successful population colonization during their northward invasion.

## 3. Discussion

### 3.1. Genetic Diversity and Large Bottleneck at the Invasion Front

High genetic diversity usually provides populations with evolutionary advantages to adapt to new environments [13]. However, introduced populations seem to always exhibit the opposite, where reduced genetic diversity relative to native populations is typically observed [4,12]. This phenomenon was also well confirmed in our results, because a large decrease in genetic diversity was observed in our invasive populations compared to the native populations. Actually, we found radical losses of genetic diversity in invasive populations, with the largest decrease in allelic diversity of 43.68–50.29% and observed heterozygosity of 52.35–53.43% at the invasive front: ZYL and WYL populations (Table 1), a degree of decline rarely observed in invasive species. Similar genetic loss of invasive *Taenioides* sp. was also observed in previous surveys based on nuclear microsatellites (SSRs) [8] and mitochondrial D-loop sequences [9]. Such losses of genetic diversity observed in invasive populations are typically attributed to a genetic bottleneck associated with founder effects: a common outcome of the establishment of new invasive populations from a small number of founding individuals [8,12]. And this has been partially verified by much higher values of genomic inbreeding coefficients (F_ROH_) observed in invasive populations than their native counterparts (Figure 1). However, despite this loss of diversity and potential limited number of founders, *Taenioides* sp. populations established and expanded successfully following their introductions, as observed in their well-documented invasive history [8,9]. This phenomenon reflects a well-explained “genetic paradox”, where invasive species are highly successful despite their initially low genetic diversity due to bottleneck effects [13]. However, high genetic diversity is not always a prerequisite for population establishment success in the invasion context. Marchini (2016) suggested that the depletion of genetic diversity induced by bottleneck events could also potentially increase population fitness by purging deleterious mutations, resulting in the production of vigorous inbred offspring [14], which allows for rapid population growth. This bottleneck-induced population fitness has also been observed in several invasive fish, such as *Serrasalmus brandtii* [15] and *Micropterus salmoides* [16]. In addition, invasive species with low genetic diversity can also benefit from rapid evolutionary changes that are favorable in the invaded regions, contributing to their overall fitness, and hence invasion success [2,7], though other factors such as ecological preadaptation of invasive species cannot yet be excluded [2,13]. Nevertheless, not all the invasive populations exhibited a radical reduction in genetic diversity relative to that of the native population. Some populations, such as GYL, exhibited well-restored genetic diversity, as observed in our analyses (Table 1). This aligns well with the comparatively long invasion history of *Taenioides* sp. in GYL, where the first spot of the invasion can be dated back to the 1980s, likely introduced as a byproduct of the Northern Jiangsu Water Transfer Project (NJWT), the precursor of the current ESNT Project which has been in place since the 1960s [8]. The restoration of genetic diversity in GYL may have arisen from multiple introductions from the source populations, which favors the accumulation of mutations and hence the genetic diversity during long-term invasion. This has made the GYL population a potential bridgehead for new water body colonization and further northward invasions. Such a genetic diversity profile and the occurrence of invasive bridgehead may have important implications in establishing where the invasive populations should be effectively controlled in the future.

### 3.2. Genetic Structure and the Invasion Source Inference

To mitigate the impact of invasive species, successful identification of the invasion source is important, which is largely dependent on the surveillance of population structure in both native and invasive ranges [3,4]. We detected a similar invasion pattern of *Taenioides* sp., as observed in previous studies based on mitochondrial [9] and microsatellite [8] datasets: the freshwater infestations in GYL were predicted to have arisen from the YE area, likely introduced as a byproduct of the ESNT project. The infestations in HZL, LML, WSL, and ZYL were likely introduced from GYL following a stepping-stone dispersal model [8]. However, previous studies were unable to achieve adequate resolution regarding the exact source of the invasion due to the marker variability, though YE has been largely implicated as the invasion source in both analyses [8,9]. Using the genome-wide approach, we clearly indicated that the native YE population was more likely to serve as a potential source for freshwater infestations than any other population. This pattern was observed in the results of our phylogenetic analyses, where all freshwater infestations aggregated in one cluster with the native population of YE (Figure 4). Such a pattern of population structure was also confirmed by our late-on structure (Figure 3) and PCA analyses (Figure 2), where the genetic composition of freshwater infestations more resembled that of YE than rest of the native populations. Though the THL population was also clustered with freshwater infestations in our phylogenetic analysis (Figure 4), its genetic composition was not closely related to invasive populations like the YE population was (Figure 2, Figure 3 and Figure 4). In addition, it also seems reasonable that THL should be expelled as the invasion source when we consider the water body connection situation and invasion history of *Taenioides* sp. in affected areas. Firstly, there are no direct water body connections between THL and the affected lakes along the ESNT route, which makes invasion unlikely via individual dispersal. Secondly, the earliest record of *Taenioides* sp. in THL could be dated back to the 1960s [17], but no freshwater invasions have ever been recorded nearby, except for some occasional spots in the connecting water bodies [18]. This may indicate that the dispersal capacity of the THL population is somewhat low, which is unlikely to underpin fast regional colonization and hence population expansion through natural diffusion in an invasion context. Other invasion sources and driving factors, such as the ESNT project must be required to facilitate the dispersal and regional colonization of *Taenioides* sp. during their northward invasion. Even under the facilitation of the ESNT project, the active dispersal of *Taenioides* sp. also seemed limited, as inferred from the substructuring of invasive populations and low gene flow among them. Similar patterns of genetic differentiation and weak gene flow were also observed among pikeperch populations in river canal systems in previous studies [19]. The low active dispersal with limited gene flow among invasive populations could partially be attributed to the deep-burrowing lifestyle of *Taenioides* sp. [10,11]. In addition, the obvious seasonality of the water transfer in the ESNT project, in which the water was mainly transferred during the late winter to spring (December to May) [20], a period that has largely missed the spawning peak of *Taenioides* sp. (June to July) around this region [21], may further constrain the active dispersal and hence gene flow among *Taenioides* sp. populations through planktonic larvae. Such source and sink relationships, and the dynamics of gene flow among *Taenioides* sp. populations may have good implications for understanding the genetic mechanisms underlying *Taenioides* sp. invasion and thus making sound measurements for mitigating the impact of *Taenioides* sp. invasion in the future.

### 3.3. Adaptive Evolution Underlying the Northward Invasions

Though our phylogenetic analyses revealed much genetic similarity between the source and invasive populations of *Taenioides* sp. (Figure 4), adaptive changes could also accumulate at the invasion front via natural selection expected from the contrasting environments they inhabit. Consistent with this inference, our McDonald– Kreitman analysis revealed a higher strength of positive selection in northernmost invasive populations compared to their lower-latitude counterparts (Table 3), despite the larger genetic bottleneck effect they may experience during the northward invasion (Figure 1). Such a genetic bottleneck effect would have resulted in a much lower selection strength in populations [22,23]. Therefore, the observed higher selection strength in northernmost invasive populations may indicate the possible adaptive selection they experience during their northward invasion, though the overall strength of the selection was somewhat low (0.101–0.136) compared with other species [24]. To identify the exact genome-wide patterns of divergence and the possible signature of adaptations in the genome during their northward invasion, our selective sweep analyses revealed substantial genomic regions and candidate genes under positive selection in WSL and ZYL populations at the invasion front (Figure 6; Supplemental Table S2). The functional enrichment analysis revealed that the top positively selected genes were significantly enriched in pathways associated with nutrient metabolism and energy production, osmotic regulation, DNA repair, and hormone production and secretion (Figure 7; Supplemental Tables S3 and S4), indicating their crucial roles in regional climate adaptations in the invasive populations. Indeed, osmotic pressure may represent an important selective force during freshwater invasion from the estuary area. Therefore, for the adaptive evolution of the genomic regions associated with nutrient metabolism and energy production, osmotic regulation would possibly contribute to the enhanced energy production and osmotic regulatory ability, which would improve fitness at the hypotonic environment [25,26] of the invasion front. However, osmotic selection alone cannot explain why there were always intervals between each colonization of the affected freshwater where no salinity gradient is available. For instance, *Taenioides* sp. has colonized GYL, LML, WSL, and ZYL in 1980, 2005, 2011, and 2014, respectively, during their northward invasion [8]. Therefore, other environmental parameters, such as thermal conditions, at the invasion front may also play important roles, as expected from the latitude gradients they span during the northward invasion. To confirm this inference, we further performed an environmental association analysis based on RDA using temperature as the variable. Our results revealed 6820 and 4667 SNPs that were strongly correlated with the two temperature variables of thetao_ltmin and thetao_mean, respectively, in sampling sites, with the majority of SNPs closely related to a lower temperature (Supplemental Figure S2). Combining both data matrices of RDA and selective sweep analyses, 1705 candidate SNPs containing 51 candidate genes were identified to be positively selected in overlap (Supplemental Table S5). These positively selected genes were primarily associated with multiple biological processes, such as metabolism and energy production (*G6PD*, *SLC25A14*, *CEL*, *PGS1*), substance transmembrane transport (*COG5*, *GOLT1B*), cytoskeletal structure and organization (*EFEMP1*, *EML6*, *TPRN*), and neural development and synaptic transmission (*SCN8A*, *CACNA2D1*, *CACNA1D*, *KCNQ2*, *SEMA3D*), implying their important roles in adaptation to regional thermal conditions during their northward invasion.

Among them, *G6PD*, *SLC25A14*, and *PGS1* are important candidates associated with mitochondrial genesis and aerobic respiration [27,28,29], while *CEL* is primarily associated with the hydrolysis of fatty acids and hence energy production [30], which may occur in thermal adaptation function. The most convincing candidate is *G6PD* for its encoding enzyme that catalyzes the rate-limiting step of the oxidative pentose–phosphate pathway, which is indispensable for maintaining the normal function of carbohydrate dissimilation and glycolysis, and hence aerobic energy production in mitochondria [29]. Mutations or abnormal expression of *G6PD* would cause severe alteration in energy production and thermal resistance in both vertebrates [31] and invertebrates [32]. In addition, adaptive alteration in *G6PD* has been observed to result in higher glycolysis catalytic efficiency and extreme low-temperature resistance in Antarctic fishes [33,34]. The apparent positive selection in these mitochondrion-associated genes in *Taenioides* sp. at the invasive front may reflect different energy requirements in adaptation to changed thermal conditions expected from the latitude gradient, although their actual role still awaits further verification. In addition, several candidate genes enriched in pathways of neural development and synaptic transmission have also been observed in invasive populations, indicating their roles in enhanced thermal adaptation and hence the success of northward invasion. This is consistent with what has already been observed in multiple teleost species, where neuronal signaling is closely related to their capacity for thermal tolerance [35,36], though the exact mechanism remains to be elucidated [37]. However, recent findings in nematode [38,39] and Drosophila [40] have begun to unravel such mysteries, and revealed that neuronal signaling could regulate the animal’s thermal tolerance through abnormal thermosensation by the thermosensory neurons, or via some neural-mediated metabolic pathways (e.g., insulin signaling), which led to altered thermal tolerance. Take *KCNQ2,* for instance: it encodes a KCNQ-type potassium channel abundantly expressed in nociceptive cold-sensing trigeminal ganglion neurons, and plays a critical role in regulating cold sensitivity in *Caenorhabditis elegans* [39]. Mutation of *KCNQ2* has been observed to cause abnormal thermosensation in ADL chemosensory neurons, and thus supranormal cold acclimation in the animal [39]. Although the close links between neuronal signaling and thermal tolerance are less well established in vertebrates, *KCNQ2* was also implicated in cold sensing and acclimation in rats [41]. Therefore, the observed positive selection in *KCNQ2* may have implications in new thermal regime adaptation at the invasion front, although the causative effect of these mutations still awaits further verification.

Interestingly, we also identified one positively selected gene (*ZP3*) that is associated with reproduction in populations at the invasion front (Supplemental Table S5). It encodes a protein at the cell surface or zona pellucida of oocytes, which are essential for species-specific gamete recognition and fertilization in reproduction [42]. However, accumulating literature has revealed that ZP3 may also play important roles in the cold tolerance of teleosts due to their binding affinity to ice, and thus potentially depressing the melting point of a solution in the cell at freezing conditions [43]. The adaptive evolution of *ZP3* has also been identified in several fish species inhabiting higher latitudes or Antarctic regions, and is largely implicated in their acclimation to the regional cold climate they inhabit [43,44]. Therefore, the obvious selection of *ZP3* in invasive populations may indicate that it also functions in regional climate adaptation in teleost species inhabiting a normal temperature regime, though, again, their actual role still awaits further verification. In conclusion, our results revealed a scenario of adaptive evolution in teleost species that underpin their regional climate adaptation and fast population colonization in a human-facilitated invasion context. Proper management strategies, such as the monitoring and physical removal of the bridgehead GYL population (e.g., using local fyke nets), or installation of exclusion structures (e.g., benthic fish screen) near the pump area, are, therefore, urgently needed to prevent their potential further northward expansion and mitigate the impact of invasion in the future.

## 4. Materials and Methods

### 4.1. Sample Collection and Genomic DNA Extraction

**Figure 8:**
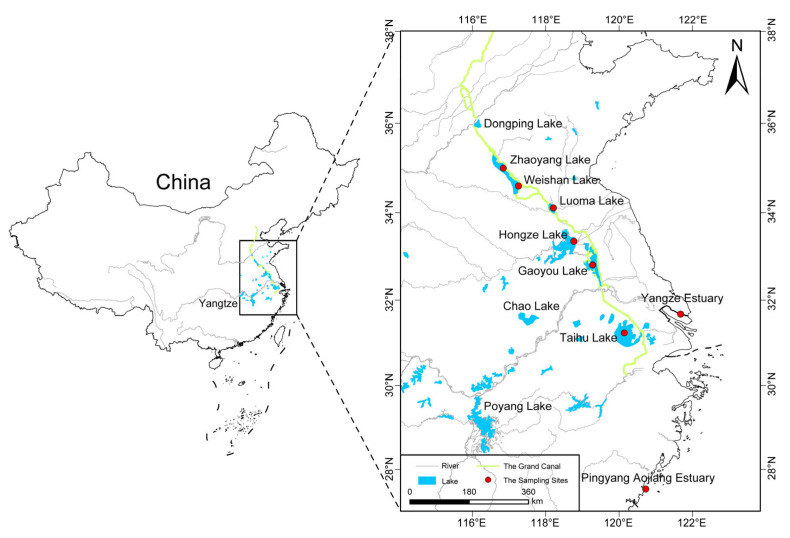
Sampling localities of invasive and native *Taenioides* sp. populations around the affected areas of ESNT projects in this study: red spots in the figure indicate the localities where *Taenioides* sp. samples were collected.

A total of 116 *Taenioides* sp. samples were collected from 5 invasive (GYL, HZL, LML, WSL, and ZYL) and 3 native populations (Yangze estuary, YE, Pingyang aojiang estuary, PYE, and Taihu lake, THL) using benthic fyke nets from April to May, 2023 (Figure 8, Table 1). Muscle tissues were collected, and the samples were stored in liquid nitrogen until use.

A Blood and Cell Culture DNA Mini Kit (QIAGEN, Hilden, Germany, cat. no. 13343) was thereafter used to isolate the genomic DNA from muscle tissues. The quality and concentration of the isolated DNA were evaluated using a Pultton DNA/Protein Analyzer (Plextech, New York, NY, USA) and stored at −80 °C prior to downstream library construction and sequencing. All the tissue sampling and DNA extraction procedures conformed to the ethical regulations formulated by the Institutional Animal Care and Use Committee of Zhejiang Ocean University (ZJOU).

### 4.2. Whole-Genome Resequencing and SNP Detection

A VAHTS Universal Plus DNA Library prep Kit (Illumina, San Diego, CA, USA) was used to construct a 150 bp paired-end sequencing library with an insertion size of 350 bp for each *Taenioides* sp. specimen. The libraries were thereby sequenced on a DNBSEQ-T7 platform (MGI Technology Co., Ltd., Shenzhen, China) with an expected target coverage of ~20×. The obtained raw sequence data were uploaded into the Sequence Read Archive (SRA) of NCBI with the BioProject accession number of JNA1290502. A Trimmomatic (version 0.39) [45] software was then used for the quality control of the raw data to remove adapters and low-quality reads with a base quality ≤ 20. The obtained clean data for each sample were then mapped to the reference genome of *Taenioides* sp. (figshare at: https://doi.org/10.6084/m9.figshare.22799645), previously assembled in our library using BWA (version 0.7.18) [46] with the parameter mem -M -k 19. The sequencing coverage and depth were then calculated using SAMtools (version 0.1.20) [47] software with the mapping data, and the potential PCR duplicates were removed using the command “MarkDuplicates” in PICARD (http://broadinstitute.github.io/picard/ accessed on 25 April 2025). SNP calling was thereafter performed using the Haplotype Caller protocol in Genome Analysis Toolkit (GATK, version 4.5.0.0) software [48], and we removed the SNPs with alow frequency of allele (MAF < 0.05) and low coverage (<10×) at the population level using GATK Variant Filtration to guarantee the reliability of the called SNPs. The remaining SNPs were then used for the downstream estimates.

### 4.3. Population Genetic Structure Inference

To infer the possible invasion process of *Taenioides* sp. within their invasive range, we first evaluated the profile of genetic diversity among populations along the route of the ESNT project by calculating the genome-wide diversity statistics (e.g., percentage of polymorphic loci (%), heterozygosity observed (Ho), heterozygosity expected (He), and nucleotide diversity (Pi)) using STACKS (version 2.68) [49]. Afterward, the genomic inbreeding based on ROH (F_ROH_) was also estimated using PLINK (version 1.9) [50] to quantify how the observed genetic diversity was attributed to inbreeding, possibly due to a bottleneck in the invasive populations. To this end, the eligible SNPs with MAF ≥0.01 were first filtered for each population. Long homozygous fragments were scanned in the pruned data using PLINK according to the following parameters: −homozyg-snp 50; −homozyg-window-missing 5; −homozyg-window-het 3; −homozyg kb 300; −homozyg-density 50; −homozyg-gap 1000; −homozyg window-threshold 0.05. The genomic inbreeding coefficient based on ROH (F_ROH_) was thereafter evaluated using PLINK according to standard methods [51]. In addition, the phylogenetic relationships and genetic structure among the populations were evaluated by clustering analyses performed using ADMIXTURE (version 1.3) [52]. To this end, sites with data missing less than 10% were used, and the number of coancestry clusters (K) was set ranging from 1 to 8. In addition, principal component analysis (PCA) was also conducted using PLINK (version 1.9) package [51] with the parameter “--−pca 10” using all SNPs. To adopt a phylogenetic perspective in the population structure inference, maximum likelihood (ML) trees were also constructed based on whole genome-wide SNPs using RAxML-NG (version 1.2.2) software [53], employing a rapid bootstrap procedure of 1000 replicates. To further estimate the magnitude of gene flow among the populations, BAYESASS 3.0 [54] was applied to calculate contemporary migration rates among populations. To this end, 90,000,000 iterations, with a burn-in of 10,000,000 iterations, and a sampling frequency of 2000 were used to ensure that the model’s starting parameters were sufficiently random. Five runs using different starting seed values were performed to ensure consistency between runs.

### 4.4. Genome-Wide Scan of Adaptive Signals

To identify the possible adaptive selection they may experience during their northward invasion, we first estimated the strength of positive selection, or the rate of adaptive substitution (α) in each population by using the classical McDonald–Kreitman test [55]. Briefly, the intraspecific diversity and divergence counts compared to the outgroup *Odontamblyopus Rebecca* (GenBank accession number: ASM3068695v1) were created from a multisequence alignment of single-copy orthologs. These alignments were first concatenated and then used as the input for imkt version 0.2 [56] to perform standard MKTs [56]. Secondly, to identify the exact genome-wide patterns of divergence and the possible signature of adaptations in the genome during their northward invasion, a combined approach involving both the Fst and genetic diversity (Pi) indexes was employed to define candidate-selected loci in the genome-wide regions in populations at the invasion front, relative to the source population. To this end, VCFtools (version 0.1.16) [57] was first used to estimate the Fst value across the genome to define candidate-selected regions using sliding windows of 100 kb (with a step size of 10 kb) if they contained window-based Fst values above the 95th percentile of the empirical distribution. Afterward, the Pi value in the same sliding windows was also calculated to identify windows above the 95th percentile of the Pi distribution. Putative selected regions were thereby identified as the overlapped windows with the top 5% of Fst and log2 (Pi) values [58]. The putatively selected regions were afterward mapped to the corresponding SNPs and genes in the genome. The enrichment of these putatively selected genes (PSGs) was also performed using Enrich GO and Rscript. A threshold of *p*-values < 0.05 was used to define the significantly over-represented GO terms and KEGG pathways.

### 4.5. Genotype–Environment Association Analyses

Water temperature may vary substantially among the lakes along the ESNT route (e.g., the monthly minimum lake surface water temperature varies from ~1.69 °C in ZYL and WSL to ~6.81 °C in YE, see Supplemental Table S1) due to the latitude gradients they span, and potentially exert large selective pressure on the *Taenioides* sp. genome. To understand the possible evolutionary changes associated with water–temperature adaptation in invasive populations of *Taenioides* sp., we further performed an environmental association analysis based on Redundancy Analysis (RDA) following the approach detailed in Dixon (2003) [59]. Briefly, we first downloaded a total of 6 water temperature variables, including monthly maximum lake surface water temperature (thetao_ltmax), monthly minimum lake surface water temperature (thetao_ltmin), annual maximum lake surface water temperature (thetao_max), annual minimum lake surface water temperature (thetao_min), annual mean lake surface water temperature (thetao_mean), and annual range of lake surface water temperature (thetao_range) in these sampling localities, which are the only water parameters available in these lakes in database of the National Earth System Science Data Center (https://www.geodata.cn accessed on 18 March 2025). To avoid theredundancy of these environmental factors, the “pairs.panels” function in R package was used to calculate the correlation among these 6 water temperature variables. For environmental factors with a correlation greater than 0.7, only one factor was retained. RDA analysis was then performed based on all the SNPs called and the preserved water temperature variables using vegan v2.6-10 program in R package [48]. To this end, the permutation was set to 999 by default, and the standard deviation was set to 3.5 (two-tailed *p*-value = 0.0005) [60] to reduce the false positive rate, and the candidate-adaptive SNPs were classified according to their highest correlation with different environmental factors.

### 4.6. Functional Annotation of Adaptive Loci

The highly selected regions detected by FST-Pi were then overlapped with the adaptive SNPs identified by RDA using the BEDtools software (version 2.31.1) [61], and only the highly adaptive regions and SNPs that were detected by both analyses were recognized as the real adaptive loci. The candidate genes corresponding to these adaptive regions and SNPs were thereby extracted based on the annotation (gff) file of the assembled genome using BEDtools. The GO and KEGG enrichment for these candidate-selected genes was then performed via Enrich GO and Rscript. Significantly over-represented GO terms and KEGG pathways were then identified with a *p*-value of <0.05.

## 5. Conclusions

In conclusion, our study demonstrated the strengths of using genomic tools to accurately estimate the role of pre- and post-introduction genetic variation, demographic history, and adaptive potential in invasion success from a genomics perspective. Using large-scale genomic re-sequencing data, we revealed that YE may serve as the invasion source, and the ESNT project serves as the main driving force facilitating their northward invasion of *Taenioides* sp. Adaptive evolution has been identified in invasive populations, despite their young invasive history, which enables their rapid range expansion, and hence their northward invasion. Regional climate, particularly the monthly minimum temperature, represented critical evolutionary forces that drive the adaptive divergence of the invasive population. Our results, for the first time, reveal a scenario of adaptive evolution in teleost species that underpin their regional climate adaptation and successful establishment of invasive populations in a human-facilitated invasion context. Our findings not only provide valuable information for accurately predicting the potential risks that hydraulic projects bring about in terms of biological invasion, but also contribute to establishing enhanced management strategies for the effective control of invasive *Taenioides* sp. in the future.

## Figures and Tables

**Table 1:** The sampling locations and the statistics of genetic diversity in *Taenioides* sp. populations within their native and invasive ranges.

Population	Sample Size	Ho	He	Pi	Polymorphic Loci (%)
ZYL	12	0.129	0.122	0.128	44.493
WSL	15	0.132	0.128	0.133	50.406
LML	15	0.133	0.130	0.134	54.124
HZL	14	0.131	0.126	0.131	51.655
GYL	15	0.181	0.174	0.180	64.380
THL	15	0.217	0.212	0.220	79.435
YE	15	0.214	0.204	0.212	77.162
PYE	15	0.277	0.278	0.288	89.498

Note: ZYL, WSL, LML, HZL, GYL, YE, THL, and anPYE represent samples collected from Zhaoyang Lake, Weishan Lake, Luoma Lake, Hongze Lake, Gaoyou Lake, Yangtze River Estuary, Thaihu Lake, and Pingyang Aojiang Estuary, respectively.

**Table 2:** Pairwise Fst values among the eight populations of *Taenioides* sp. within their native and invasive ranges.

Population	ZYL	WSL	LML	HZL	GYL	THL	YE	PYE
ZYL								
WSL	0.021							
LML	0.029	0.024						
HZL	0.030	0.025	0.018					
GYL	0.056	0.053	0.047	0.049				
THL	0.075	0.074	0.070	0.072	0.054			
YE	0.057	0.056	0.052	0.053	0.031	0.035		
PYE	0.130	0.097	0.094	0.095	0.074	0.055	0.052	

Note: ZYL, WSL, LML, HZL, GYL, YE, THL, and PYE represent samples collected from Zhaoyang Lake, Weishan Lake, Luoma Lake, Hongze Lake, Gaoyou Lake, Yangtze River Estuary, Thaihu Lake, and Pingyang Aojiang Estuary, respectively.

**Table 3:** Proportion of adaptive amino acid substitutions (α) estimated for different *Taenioides* sp. populations with standard McDonald– Kreitman tests (MKTs).

Population	α-Value	Fisher *p*-Value
ZYL	0.135	3.17 × 10^−21^
WSL	0.126	2.14 × 10^−20^
LML	0.136	6.68 × 10^−25^
HZL	0.123	1.36 × 10^−19^
GYL	0.101	1.43 × 10^−16^
THL	0.099	4.56 × 10^−19^
YE	0.120	8.54 × 10^−28^
PYE	0.149	1.23 × 10^−52^

## Data Availability

All raw genome re-sequencing data for *Taenioides* sp. genome are deposited at the NCBI in the sequence read archive (SRA) under accession number BioProject number: PRJNA1290502.

## Supplementary Materials

The following supporting information can be downloaded at: https://www.mdpi.com/article/10.3390/ijms26199613/s1.
