# Supplementary material for: Genomic Signatures of Adaptive Evolution in Taenioides sp. During Northward Invasion
Source: Int J Mol Sci. 2025 Oct 1;26(19):9613. doi: 10.3390/ijms26199613 (PMC12525211; doi:10.3390/ijms26199613)
Supplement: Supplementary file 1 [file ijms-26-09613-s001.zip › Supplementary+figures.pdf]

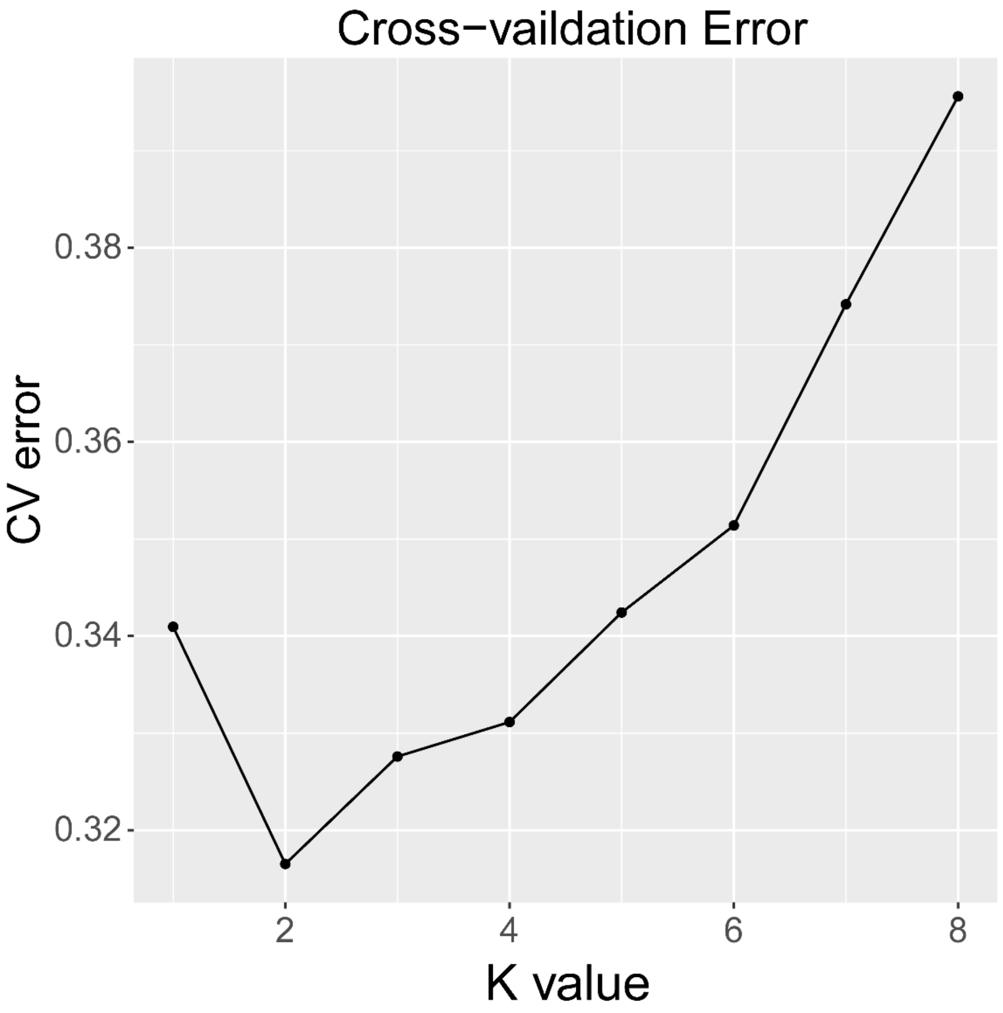

Figure S1. Cross-validation (CV) errors under different K values. lower CV errors indicate higher reliability of population structure.

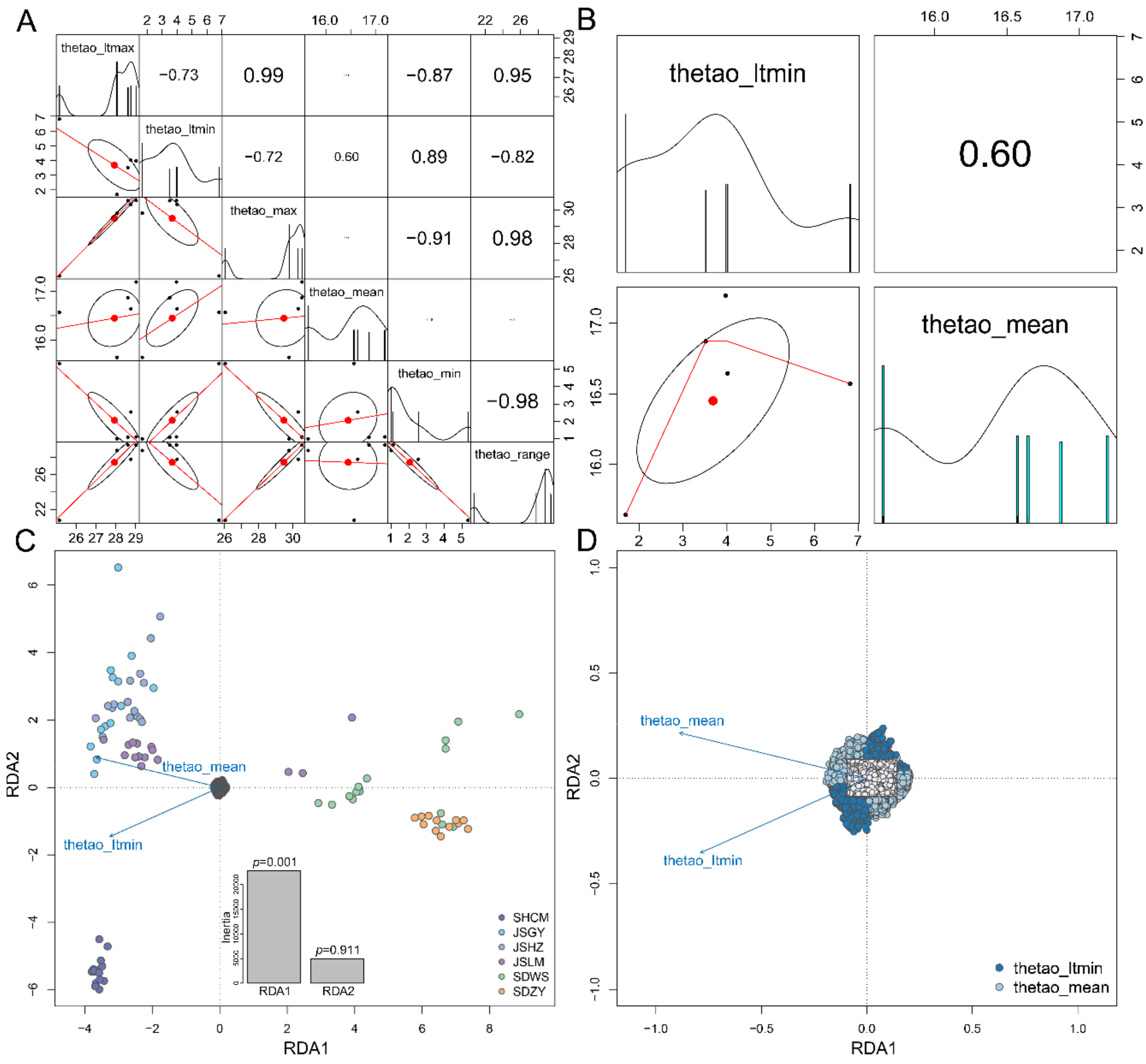

Figure S2. (A) The results of environmental factor correlation and redundancy analysis (RDA). Environmental variable correlation matrix diagram, showing the pairwise correlation between the 6 temperature variables. The diagonal line in the figure is the distribution frequency histogram of each variable; the lower triangle is the scatter plot and fitted trend line between the variables; the upper triangle is the Pearson correlation coefficient between the variables. (B) Based on an empirical threshold (|r| > 0.7), highly collinear environmental variables were removed, and only thetao_ltmin and thetao_mean were retained for subsequent RDA analysis. (C) Redundancy analysis (RDA) on axes 1 and 2 performed with the all SNP loci called. Blue vectors represent environmental predictors. thetao_ltmin, and thetao_mean represent monthly minimum lake surface water temperature and annual mean lake surface water temperature, respectively. The dark grey cloud of points at the centre represents the SNPs, and the coloured points represent individual *Taenioides sp* of different sampling locations. (D) Magnification of to highlight SNP loadings on axes 1 and 2. Candidate SNPs are shown as coloured points with coding by the most highly correlated temperature predictor. SNPs not identified as candidates (neutral SNPs) are shown in light grey.
